# Age, gender, and the fear of getting Alzheimer’s disease

**DOI:** 10.1192/j.eurpsy.2023.1440

**Published:** 2023-07-19

**Authors:** M. Bystad, N. R. Aas, V. Sollid, R. Wynn

**Affiliations:** 1 Division of Substance Use and Mental Health, University Hospital of North Norway, Tromsø; 2 Oslo University Hospital, Oslo; 3 Department of Psychology; 4 Clinical Medicine, UiT The Arctic Univeristy of Norway, Tromsø, Norway

## Abstract

**Introduction:**

Alzheimer’s Disesase is the most frequent cause of dementia, accounting for approximately 60% of cases. It is characterized by an accumulation of beta amyloid and tau protein in the brain, resulting in the loss of normal brain tissue and cognitive decline, including loss of memory and language. Prior studies have found that this is one of the most feared disorders, possibly because of the associated cognitive decline, our poor ability to prevent and treat the disorder, and its poor prognosis. Prior studies have found different results regarding the importance of age and gender on level of fear.

**Objectives:**

We wanted to study the fear of obtaining Alzheimer’s disorder in a Norwegian sample and to examine the importance of age and gender.

**Methods:**

The Fear of Alzhemer’s Disease Scale (FADS, French et al, Geriatr Psych 2011;27:521-8) was translated into Norwegian for this study, following standard procedures. The questionnaire has 30 items, each responded to on a 5-point likert scale with responses ranging from ‘never’ to ‘always’. The total maximum score was 120 points. Links to the questionnaire were posted on Facebook. Respondents were directed to a site for anonymous and untracable participation. SPSS version 24 was used for statistical analyses. Non-parametrical tests, including the Mann-Whitney U-test, were used to study between-group differences (age below 50/others, male/female).

**Results:**

The FADS score was significantly higher (U=5113, Z=-2.236, p=0.025) in the respondents below 50 years (60.00) than in the others (54.93). The FADS score was not significantly different (U=7513, Z=1.673, p=0.094) between men (56.12) and women (59.67).

**Conclusions:**

We found that the level of fear, on average, was quite high. Those below 50 years were significantly more fearful of the disorder than the older respondents. This might seem counterintuitive, as the disorder is much more common in older people. However, emotional regulation and fear of illness may improve with age (Carstensen et al. Psychol Aging 2011;26:21-33), which might explain our finding. There was no significant gender-related difference in fear of getting Alzheimer’s Disease, which is interesting given that 2/3 of those suffering from the disorder are women. The study was based on a questionnaire posted online, which might have resulted in a bias in participation. Further studies are needed to confirm our findings.

**Disclosure of Interest:**

None Declared

